# Living in an oasis: Rapid transformations, resilience, and resistance in the North Water Area societies and ecosystems

**DOI:** 10.1007/s13280-018-1034-y

**Published:** 2018-03-08

**Authors:** Erik Jeppesen, Martin Appelt, Kirsten Hastrup, Bjarne Grønnow, Anders Mosbech, John P. Smol, Thomas A. Davidson

**Affiliations:** 1 Department of Bioscience, Aarhus University, Vejlsøvej 25, 8600 Silkeborg, Denmark; 2 The National Museum of Denmark, Frederiksholms Kanal 12, 1220 Copenhagen K, Denmark; 3 Department of Anthropology, University of Copenhagen, Øster Farimagsgade 5, 1353 Copenhagen K, Denmark; 4 Department of Bioscience, Arctic Research Centre, Aarhus University, Frederiksborgvej 399, 4000 Roskilde, Denmark; 5 Department of Biology, PEARL, Queen’s University, Kingston, ON K7L 3N6 Canada

**Keywords:** Fast transformations, High Arctic, North Water, Regime Shifts, Resilience, Resistance

## Abstract

Based on lake sediment data, archaeological findings, and historical records, we describe rapid transformations, resilience and resistance in societies and ecosystems, and their interactions in the past in the North Water area related to changes in climate and historical events. Examples are the formation of the polynya itself and the early arrival of people, ca. 4500 years ago, and later major human immigrations (different societies, cultural encounters, or abandonment) from other regions in the Arctic. While the early immigrations had relatively modest and localised effect on the ecosystem, the later-incoming culture in the early thirteenth century was marked by extensive migrations into and out of the area and abrupt shifts in hunting technologies. This has had long-lasting consequences for the local lake ecosystems. Large natural transformations in the ecosystems have also occurred over relatively short time periods related to changes in the polynya. Finally, we discuss the future perspectives for the North Water area given the many threats, but also opportunities.

## Introduction

The Arctic is facing major shifts in the sea-ice system, and this is also true for the North Water polynya although changes are not straight-forward. The North Water area includes the lands on the Greenland side bordering the polynya from Cape York to the Humboldt Glacier, and on the Canadian side, the lands south of Fort Conger to the northeastern side of Lancaster Sound. In recent years, glaciers and icecaps have been portrayed as canaries in coal mines and become part of a growing set of narratives about climate change that are highly relevant when it comes to address the challenges to humans and nature in the near future (Daniels and Endfield 2009). The metaphor of the canary is understandable, but it may potentially skew the issue in the public mind by presenting the climate-change indicators as “lone voices, single examples of a species or object, presented in emotive isolation from their ecological contexts” (Hamblyn 2009, p. 231). Whether the ‘voice’ belongs to the glacier or the polar bear, there is a risk of forgetting how components of the ecosystem connect and make up comprehensive wholes of interdependent elements and histories. The implicit repression of complexity is often sustained by a particular visual strategy, for instance an image of a skinny polar bear or, indeed, an indigenous Arctic hunter, jumping from floe to floe in dramatic pursuit of his livelihood (Martello 2008). The far more complicated issue is to show how the fates of polar bears and indigenous hunters are tied up with multiple species, long histories, and natural processes of many kinds and temporalities, and with pressing issues of governance and power—all of which will be addressed in this article.

While changes in both ecosystems and societies may be gradual, it is also evident that major shifts have occurred in the North Water area at different times. In this paper, we seek to identify and discuss possible relations between “fast” transformations in ecosystems and decisive historical “events” that substantially changed the historical trajectories of human societies living around the North Water in the past. We also discuss drivers (external, internal or a combination of the two) of major changes in the North Water area.

Within traditional, natural scientific ecosystem thinking, concepts such as “transformations”, “tipping points”, and “alternative states” are generally seen as driven by external perturbations (Box 1). In addition, the concepts of “critical transitions” and “tipping points” are warranted only if these states are generated by a gradual change in one or more key drivers leading to a fast and all-encompassing transformation in a more or less stable “alternative state” of the ecosystem.

**Box 1:** Defining regime shifts

**Fig. 1:**
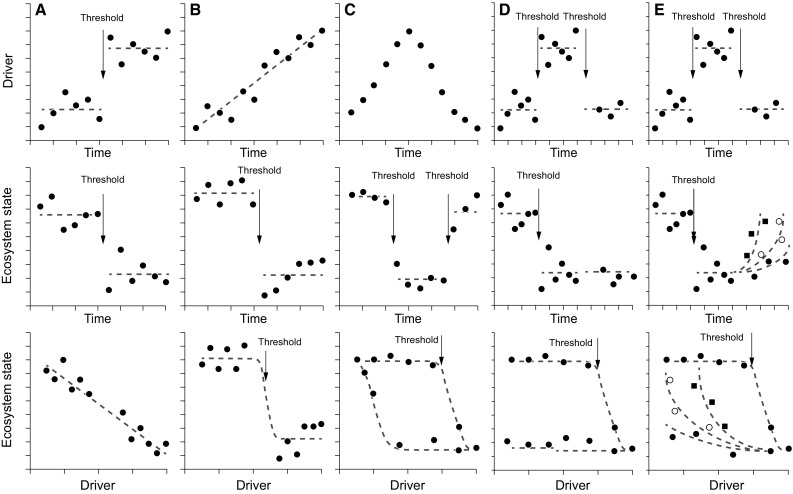
Differences between regime shifts resulting from smooth pressure–status relationships, threshold-like responses, and alternative stable states (bistability) with hysteresis. The two top rows of graphs show time series of driver (e.g., nutrient inputs) and ecosystem state (e.g., phytoplankton biomass), and the lower row of graphs show the relationship between the driver and ecosystem state. **a** Regime shift in driver is linearly translated to the ecosystem state. Jumps appear only in the time series. **b** Regime shift in ecosystem state after driver exceeds a threshold (tipping point). This is manifested through a jump in the time series of the ecosystem state. **c** The hysteresis loop linking the ecosystem state to the environmental driver results in jumps between two alternative states when the driver is first slowly increased and then reduced again. **d** Regime in drivers leading to changes in ecosystem state that remain (or change slowly back, not shown) when the former driver situation is re-established. **e** Regime in drivers leading to changes in ecosystem state that change gradually back (with differential resilience illustrated with different symbols) when the former driver situation is re-established. **a**–**c** Taken from Andersen et al. (2009)

Ecological regime shifts can be defined as abrupt transformations (regime shifts) leading to ecosystem reconfiguration. These shifts are generally thought to be driven by external perturbations (e.g., climatic fluctuations, overexploitation, eutrophication, etc.). There are at least five ways in which an ecological system can exhibit abrupt changes over time; two are directly reversible in response to changes in environmental drivers, and three show resilience of different degree (Fig. 1). An abrupt shift may occur if the key driver(s) show abrupt shifts or it may occur with gradual changes in drivers when a tipping point is reached. In the latter and only the latter case, we can talk about a critical transition, a term that has become misused across disciplines. Thus, an abrupt change is a necessity, but not a sufficient condition for demonstrating critical transitions and defining tipping points, as the change might simply be driven by sudden changes in the main drivers of the systems	

The stability of the “alternative state” is coupled with concepts such as “resistance” and “resilience”, which, again, among others, has to do with the sensitivity to changes in other external drivers and possible internal buffers (Box 2).

**Box 2:** Stability concepts in ecology. In this box, we describe the most commonly used stability concepts, their properties, and how to measure them (based on Grimm et al. 1992)

Stability concept	Definition of the related stability property	Related measures
Constancy	Staying essentially unchanged	Standard deviation, annual variability
Resistance	Staying essentially unchanged despite the presence of potentially disturbing external influences (disturbances)	Sensitivity, buffer capacity
Resilience	Returning to the reference state (or dynamics) after a temporal external influence (disturbance) has been applied	Return time, size of domain of attraction
Persistence	Persistence through time of population (or other ecological units based on populations)	Mean time to extinction, value of a lower nonzero limit of the state variable

In ecosystem population and system constancy, resistance and resilience are expected to be stronger in spatially heterogeneous systems that allow alternative feeding possibilities and refuges of populations and greater buffer conditions against pressures on the ecosystems at large.

Moreover, scales matter: large areas with higher heterogeneity are usually more resilient and have lower risk of major fluctuations (Turner et al. 2013). Furthermore, systems that are frequently subjected to major disturbances are often more resilient (showing faster recovery) than those subjected to less frequent catastrophic events (Seidl et al. 2014). Ecosystems with a high degree of connectivity to adjacent ecosystems are also expected to be more constant and resilient (Timpane-Padgham et al. 2017), though high openness also poses an enhanced risk of (sometimes unwanted) species invasion, some of which might potentially change the ecosystems substantially.

In archaeology and history, *historical events* are understood as sudden, often unpredictable, events that retrospectively can be seen to have made considerable impact on people and other living beings. Such events may be the result of *historical processes*, implying a more gradual build-up to the point where sudden and irreversible social and economic changes took place. The changes may be both internally and externally induced, even if this distinction does not always make sense in an open-ended world. Below, we shall discuss some examples of events and processes that have led to turning points in history.

The concepts of “historical events” and “historical processes” are not directly translatable to the natural science concepts discussed in the present article. However, we want to suggest that human histories around the North Water area include circumstances in which it is relevant to use the concepts of critical transitions and tipping points in trying to understand the mutual impacts of human and non-human history. In addition, we find it possible to identify several examples of buffers, such as secondary biotic resources and different types of cash-crops that have sustained resilience across different historical periods. Such buffers help stabilising the human population, as overexploitation of resources may be avoided and ecosystem damage with potential loss of basal resources for humans minimised. Likewise, a degree of openness, including migrations of people and animals, may also help sustaining populations, while also carrying potential risks, with new populations taking over or overexploiting resources due to imported new and more efficient technologies, for instance firearms and motorboats, unless regulations are established. This goes to suggest that socio-ecological systems may be analysed along the same lines as ecosystems generally. What differs is the nature of the system, the socio-ecological system always including humans and their capacity for strategic thinking, innovation, and collaborative deliberation.

In the social sciences, resilience conventionally points to the amount of perturbation that a particular society or community can absorb and still be recognizable, also to itself. In other words, resilience is “the capacity to change in order to maintain identity” (Carmack et al. 2012, p. 60). In Arctic social systems, this calls for a high degree of flexibility (Hastrup 2009)—defined by Gregory Bateson as ‘uncommitted potential for change’ (1972, p. 497). When flexibility is no longer possible, because all energies are committed to bare survival, the system may reach a tipping point – “where relatively small perturbation can cause a large, qualitative change in the future state of the system” (Wassmann and Lenton 2012, p. 3) (Box 1, Fig. 1b scenario). In social communities, regime shifts always imply a degree of human agency, but the term may still be used productively, provided that the essential human capacity for conscious acting is acknowledged and the impossibility of an absolute ‘return’ to the previous states implicitly recognized.

## Examples of fast transformations, resistance, and resilience in the north water area

There are many examples of fast transformations and differential degrees of resilience and resistance in the societies and ecological systems as well as in interactions between societies and ecosystems—embraced in the term socio-ecological system, to which we shall return later. In this section, we provide some examples from the North Water area.

### The Late Dorset and Inuit in the NOW Polynya (13th to 15th centuries AD): Whales and driftwood as drivers of regime shifts

**Fig. 2:**
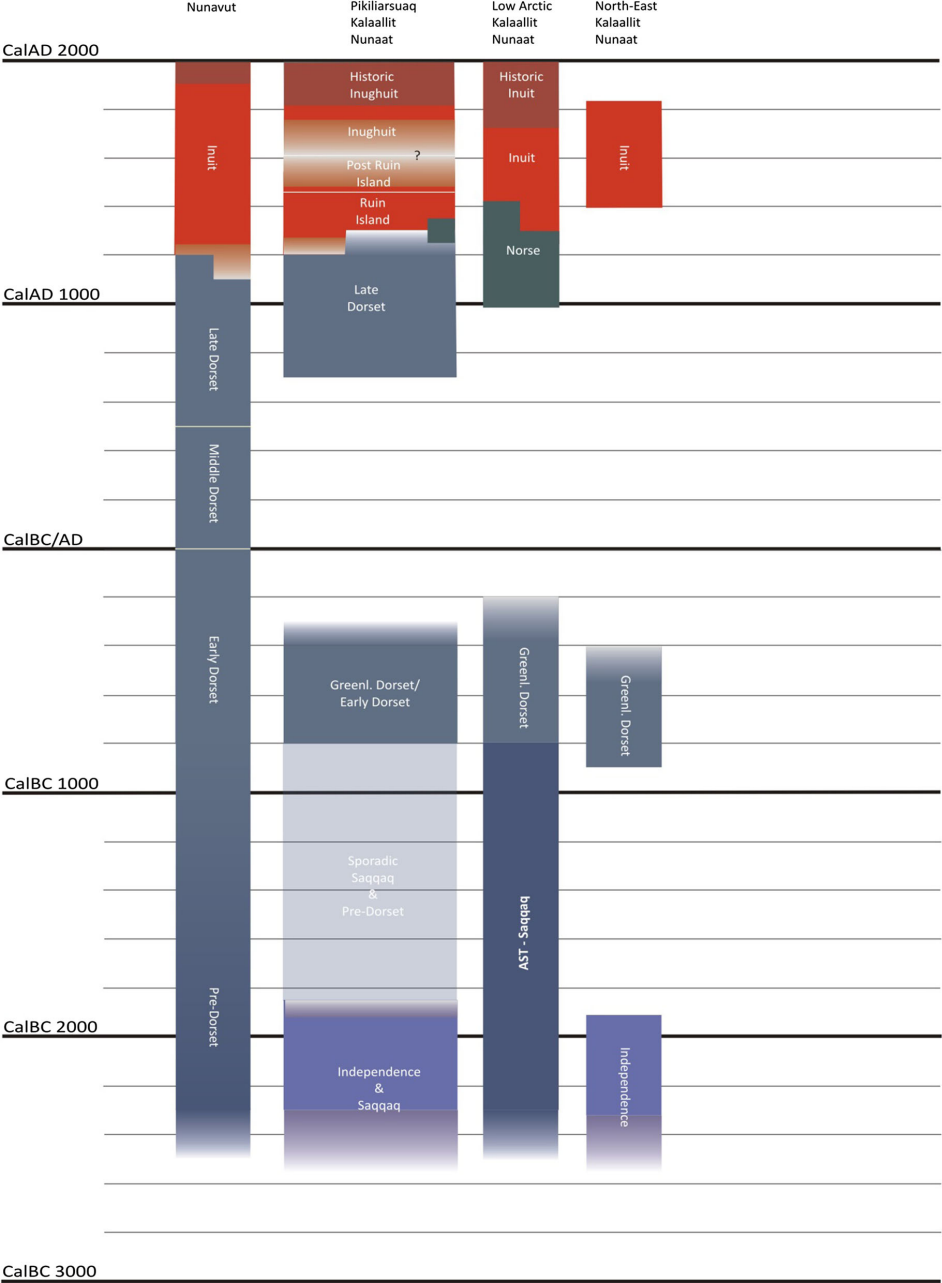
Cultural chronology of Eastern Canada (Nunavut) and Greenland (Kalaalliit Nunaat). The second column from left (Pikiliarsuaq) covers the cultural chronology of the North Water area. The processes behind the noticeable discontinuities in human presence in various parts of Greenland are not well understood. The chronological scheme is a modified version of the scheme by Appelt, Friesen, and Grønnow in Raghavan et al. (2014)

A number of encounters between very different people in the North Water area (Fig. 2) in the 13th and 14th centuries are crucial for our understanding of the particular historical processes across all of Greenland (Appelt and Gulløv 2009).

It was during these centuries that Alaskan Inuit groups crossed the Mackenzie Delta and settled the eastern Arctic. It has been suggested that one of the most important drivers was the unique source of iron located in northern Melville Bay, i.e., the famous Cape York meteorites (McGhee 1984, 2009). Judging by the layout of the early Inuit sites, the groups arriving were organised around a whale-boat owner (an *umialik*), his crew, and their families. The winter sites generally had 3–8 winter dwellings (although larger numbers have been found) built from a combination of sod, boulders, and slabs, and various baleen whale bone elements. The dwellings had a separate kitchen niche containing an open fireplace. Most would have housed one or two nuclear families, but many of the sites had a separate, larger and different type of dwelling structure—the so-called Men’s houses, or *qassi* (Holtved 1944, 1954; McCullough 1989). The finds from these Men’s houses often include substantial amounts of baleen, and besides housing various social functions, they seem to have functioned as workshops (Mønsted 2016). With the arrival of the Alaskan Inuit, we have the first evidence of the large seagoing vessels known as *umiat* (sing. *umiaq*) in the eastern Arctic. These boats were not only used as whaling boats but could carry considerable gear and about 10–12 people. *Umiaq* construction demanded a relatively large amount of high-quality wood, and maintenance required an annual renewal of the skin cover, made from either bearded seal or female walrus (Petersen 1986).

Judging by the radiocarbon dates available from the North Water area, there is little evidence of Men’s houses after the middle of the fifteenth century, and no direct evidence of the *umiaq* is known from the area. Thus, it is likely that the *umialik* institution disappeared from the North Water area at this time when also the ordinary winter dwellings lost their separate kitchen to be replaced by small niches in which the source of heat was blubber lamps. It thus seems likely that considerable social and economic transformations (a regime shift, Box 1, Fig. 1a or b scenario) took place in the fifteenth century. An important driver in these changes would have been a gradual exhaustion of the wood brought from Alaska and the local and limited sources of driftwood, owing to the expanding ice cover in the Baffin Bay during the Little Ice Age (Alix 2016; Hastrup et al. 2018). Even if we do not have a sufficiently refined chronological control of the order of events that took place in prehistoric times, it does seem clear that a substantial amount of Inuit moved further south in Greenland to settle all areas from south of the Melville Bay to Cape Farewell, and then north into East Greenland during the late thirteenth and early fourteenth century. From the early part of the fifteenth century, other groups of Thule Inuit moved northwards around Peary Land and then south into Northeast Greenland (Gulløv 2004; Sørensen and Gulløv 2012).

When the Alaskan Inuit first arrived in the North Water area (Fig. 2), it had been inhabited since the eighth century by the Late Dorset people, as they are known in archaeology. The timing and character of the meetings between the Inuit and Late Dorset are still strongly debated (e.g., Friesen 2000; Appelt et al. 2016; Park 2016), but clearly the social organisation and technologies of the two groups were very different. The incoming Alaskan Inuit groups were in control of the long-distance exchange networks, from the fourteenth century onwards, including the exchange of meteoric iron to most parts of the Canadian Arctic (Appelt et al. 2016). By this time, we see the complete disappearance of the Late Dorset groups, not only in the North Water Area but throughout the eastern Arctic (Appelt et al. 2016). It seems likely that their disappearance is in part the result of the arrival of the Inuit (Friesen 2000; Appelt 2004), and it has been suggested that the Alaskan Inuit may have—unknowingly—exposed the Late Dorset groups to diseases to which their immune system had no defence, i.e., in epidemiological terms, the Late Dorset groups would have been a “virgin population” (Agger and Maschner 2009) (Box 1, Fig. 1a scenario).

A third party was involved in these meetings, namely the Norse. We do not know what the frequency and extent of these meetings were, but we do know that they took place sometime in the late thirteenth and fourteenth century and that Norse artefacts from these meetings are distributed across at least nine different Inuit sites (Holtved 1944, 1954; Schledermann 1980; McCullough 1989; LeMoine and Darwent 2010; Grønnow et al. 2016) and one of the Late Dorset sites (Appelt et al. 1998). The Norse presence in the North Water area would have made it clear to the Alaskan Inuit groups that they had to go further south for supplies of, among other items, wood and iron.

For the Late Dorset groups, the arrival of the Inuit meant that they and their lifestyle vanished—a rapid transformation within a century. For the Inuit arriving in the region of the North Water area, it opened numerous rich and diverse resource spaces allowing them to continue most of their Alaskan lifestyle. However, after just one or two centuries, this would no longer have been viable. A major driver in this process is likely to have been a gradual exhaustion of wood supplies, as mentioned above, and/or a change in the timing and migration routes of the large whales in the North Water, which could be due to the onset of the Little Ice Age by the early fifteenth century. The situation was, however, buffered by moving into new lands—further south—which they came to fully utilise after the disappearance of the Norse in the middle of the fifteenth century.

### Effects of Thule Inuit whalers on the environment

While we have little data from the North Water area on the effects of Thule Inuit on the local ecosystems, paleolimnological and archaeological data collected from the Canadian Arctic suggest that the whaling activities of the Thule Inuit altered freshwater ecosystems markedly. For example, using paleolimnological approaches, Douglas et al. (2004) and Hadley et al. (2010a, b) revealed that the Thule winter settlement on Somerset Island, south-eastern Bathurst Island, and on east-central Ellesmere Island (Nunavut, Arctic Canada), respectively, led to marked ecological changes in pond ecology due to excess nutrient input (eutrophication) from the Thule Inuit activities.

**Fig. 3:**
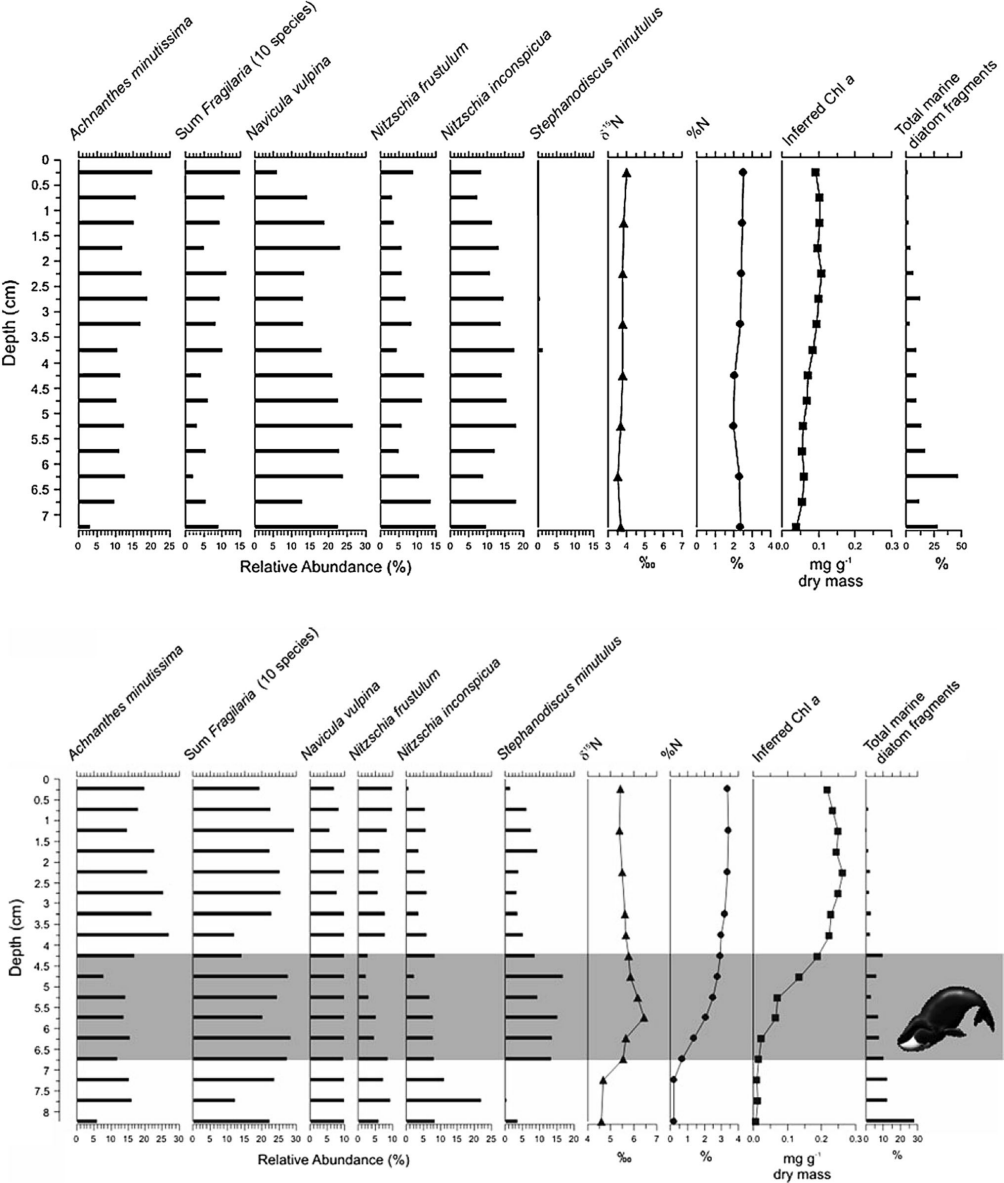
Using a comparative approach to demonstrate the effects of Thule Inuit winter settlers on ponds in Arctic Canada based on algal remains in sediment cores. The profiles represent diatom species composition, δ^15^N, %N, and spectrally inferred chlorophyll *a* from two similar ponds on Bathurst Island, only a few metres apart. However, Pond B-AO (above) had only 5 Thule whalebone houses in its catchment, whilst Pond B-AP (below) had 16 houses. The more affected pond clearly documents the limnological response of the whalers. For example, the diatom *Stephanodiscus minutulus* is a well-known indicator of elevated nutrient conditions (modified from Hadley et al. 2010a)

Using a comparative approach, Hadley et al. (2010a) recorded striking ecological changes in diatom species assemblages, spectrally inferred primary production and nutrient geochemistry, indicating nutrient enrichment and eutrophication in a small pond draining 16 Thule Inuit whale houses, whilst an adjacent pond with only five whale houses recorded only minimal changes (Fig. 3). Input of marine-derived nutrients from sea mammal carcasses used by the Thule Inuit for food and construction of winter settlements, and other human activities, coincided with a notable increase in a eutrophic diatom taxon. Although the diatom changes recorded in the affected site persisted for some time after the period and Thule occupation, indicating relatively strong resilience, the most recent sediments and water chemistry suggest that the pond has now, however, partly recovered to near pre-impact conditions (Hadley et al. 2010a) (Box 1, Fig. 1e scenario).

The effect of the Dorset culture on the pond ecosystem can also be recorded in the paleolimnological record. Michelutti et al. (2013) analysed sediment cores from seven ponds on the south-western coast of Baffin Island, Nunavut, covering a gradient of the intensity of past human activity in their catchments. The study area was, prior to the historic Inuit occupation, extensively inhabited by Thule culture Inuit (ca. 12–16th century) and by an earlier Arctic group, the Dorset culture Palaeo-Eskimos (ca. 500 BC—fifth century) and their predecessors from a period as early as 2500 BC. The authors found that the degree of eutrophication in these freshwater ponds depended on the length of the occupation as well as the amount and type of marine mammals taken as primary prey items (e.g., whales, walrus, or seals).

**Fig. 4:**
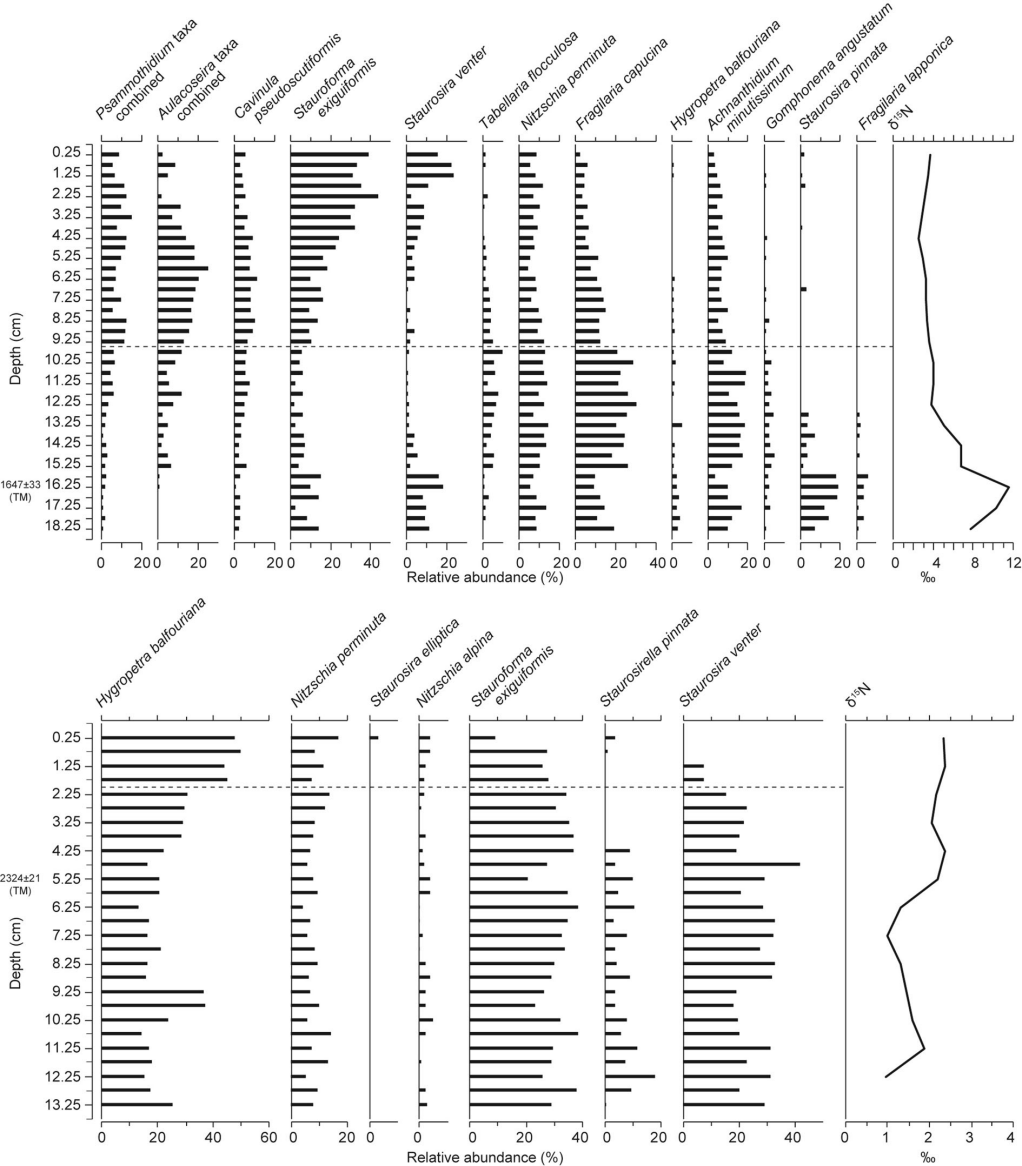
Above: Effects of the Dorset sealing activities on a pond on Baffin Island (Nunavut, Arctic Canada) based on diatom assemblage structure and sedimentary δ^15^N. The effects of Dorset sealing activities are recorded near the base of the core. The primary diatom zonation, as determined by CONISS (Grimm 1987), is shown as a dotted line. The radiocarbon date, shown on the leftmost side, was obtained on terrestrial macrofossils (TM) (Modified from Michelutti et al. 2013). Below: The fossil diatom stratigraphy and profile from Juet-1 on Baffin Island (Nunavut, Arctic Canada), a pond adjacent to a Dorset longhouse, believed to be used primarily for periodic seasonal gatherings and ceremonial functions. The relatively complacent profiles indicate no major human impacts at this site, as would be expected. The primary diatom zonation as determined by CONISS (Grimm 1987) is shown as a dotted line. The radiocarbon date, shown on the leftmost side, was obtained from terrestrial macrofossils (TM) (Modified from Michelutti et al. 2013)

Similar to the Thule examples noted above, the limnological effects of Dorset sealing activities were clearly demarked in the sediment record. For example, Tanfield-1 is a pond adjacent to a Dorset archaeological excavation and the sediment record clearly identified the effects of past sealing and other Dorset activities by elevated δ^15^N and changes in diatom assemblages near the base of the core (Fig. 4, above). A nearby control pond (Tanfield-2), outside the influence of the Dorset, recorded no similar changes (Michelutti et al. 2013). Meanwhile, sediment cores collected near the so-called Dorset “longhouses”, such as Juet-1 (Fig. 4, below), believed to be locations of periodic seasonal gatherings, did not register any evidence of eutrophication, reflecting the shorter, less intensive nature of these occupations. Due to slow rates of decomposition, nutrients from butchered marine animal bones continue to influence the freshwater sites into which they drain, as evidenced by higher than typical nutrient- and production-related water chemistry variables (Douglas et al. 2004; Hadley et al. 2010a, b; Michelutti et al. 2013). These examples show the mutual impact of the partners in what emerges as a truly *socio*-*ecological* system.

### Historical drivers and resistance, the story of Qillarsuaq

Apart from the environmental drivers of change, human, social, or cultural factors must also be taken into account—as we saw in the example above. In an article on population movements in the Canadian Arctic, the Canadian anthropologist Susan Rowley (1985) provides a systematic analysis of 25 historically and/or orally documented large-scale Inuit population movements. In six cases, the driver behind the movement is likely either famine or epidemic diseases (Box 1, Fig. 1a or b scenario, depending on whether these diseases appeared once or got gradually worse), and in a seventh case a natural disaster. Rowley suggests that in 10 cases, the impetus has to be found in what she calls social pressures, chief among which was fear of revenge.

The best documented case of direct relevance to our study area is that of the travels of the famous Qillarsuaq, who eventually had a major effect on the lives of people in the North Water area (Gilberg 1976; Ulloriaq 1985; Mary-Rousselière 2002; see also Hastrup et al. 2018). Qillarsuaq’s long journey seems to have begun with a killing in southern Baffin Island in the early 1840s. Having lived for a while in the Pond Inlet region, Qillarsuaq got into a conflict during which he committed a second murder, and after fleeing, he headed north and eventually joined up with Inughuit in Inglefield Land in the early 1860s. Some 6 years later, he decided to leave Greenland, after having taken part in the killing of a powerful local hunter and angakok (”shaman”). Qillarsuaq died shortly after leaving Greenland.

Even though this case revolves around Qillarsuaq, it originally included some 50–60 people leaving Pond Inlet, of which the larger part died during the journeys across rarely travelled parts of Devon Island and Ellesmere Island. The historical sources do not provide an insight into whether or not gradually increasing social tensions had been building in Pond Inlet prior to Qillarsuaq’s arrival, but the resulting social and economic effects on the community in Pond Inlet must have been substantial as the movement included an estimated quarter or third of the total population in the area. It is, however, clear that the arrival of the group led by Qillarsuaq resulted in significant changes in the life among the Inughuit. The Baffin Island group (re-)introduced a number of technologies—the kayak, the *umiaq*, the use of bow and arrow, and of fishing hooks and leisters—that resulted in a dramatic expansion of the resource base of the Inughuit. Only a decade earlier, prior to the arrival of the group, serious concerns about the survival of the Inughuit were expressed by both British explorers in the area and by indigenous people themselves, with, perhaps, the entire population amounting to 100–140 persons (Kane 1856; Hayes 1867, quoted in Gilberg (1976)). Just a generation later, due to the immigration of the Baffin islanders, the population seems to have increased to a more viable minimum at about 220–250 people (Gilberg 1976), thus being an example of openness creating a more robust, or resilient, community. Given his brief sojourn, it was, indeed, a fast transition.

Prior to Qillersuaq, the arrival of John Ross and his crew in 1818 also made a great impact in the Thule region; the arrival marked the first historically known meeting between Europeans and Inughuit (Ross 1819). Ross noted that the Inughuit did not have any kayaks or *umiaqs* nor possessed knowledge of how to build these. His interpreter, Sakæus, was told that the knowledge was lost following a series of deaths of the people who knew how to build them many years earlier. He was also told that the Inughuit had no contact with other people, either to the south or to the west. Sakæus was surprised to see that dog sledges were made of bone and antler without the use of wood. These examples follow a Box 1, Fig. 1e scenario. Following immediately in the wake of Ross, the North Water and Inughuit were visited by primarily Scottish whaling ships that not only managed to wipe out the entire baleen whale population in the North Water by the turn of the twentieth century but also (along with British explorers) brought an (uneven) stream of European products into the area (Gilberg 1976).

It is highly likely that the very low population number recorded in the 1850s and 1860s was a direct result of various diseases introduced by Europeans into the, in epidemiological terms, “virgin” Inughuit population, as we see in later more well-documented cases. Historical sources thus suggest that 10 to 20% of the Inughuit population died from each of a series of epidemics starting around 1880, and occurring in 1895–96, 1901–02, 1909–10, 1920–21, 1928–29, 1932, 1945, 1948–49, and 1955 (Gilberg 1976, p. 27–30).

When combining the information contained in the records on the Inughuit community, it is plausible that the main drivers in the erosion of the Inughuit society in the early nineteenth century were epidemics and isolation. While the lack of several technologies reduced the resource spaces available to the Inughuit community, this seems to have been overcome by intensifying the harvest of an otherwise secondary resource, Little Auk (*Alle alle*), thus acting as a buffer. The widening of resource spaces with the arrival of Qillarsuaq and his group seems to have been sufficient to act as a resistance preventing the passing of a tipping point that would, otherwise, have led to the extinction of the Inughuit.

### The arrival of the Little Auk transformed the local ecosystems

The Little Auk was not only a food buffer for local people, creating social resilience and preventing extinction; it was also a key ecosystem engineer shaping the terrestrial and freshwater ecosystems along the Greenlandic coast in the North Water area and elsewhere within its breeding range. The Little Auk promotes the assimilation of marine-derived nutrients in terrestrial and aquatic biota and boosts primary productivity, which in turn has profound effects on a range of biogeochemical processes in the ecosystems involved (González-Bergonzoni et al. 2017; Mosbech et al. 2018).

**Fig. 5:**
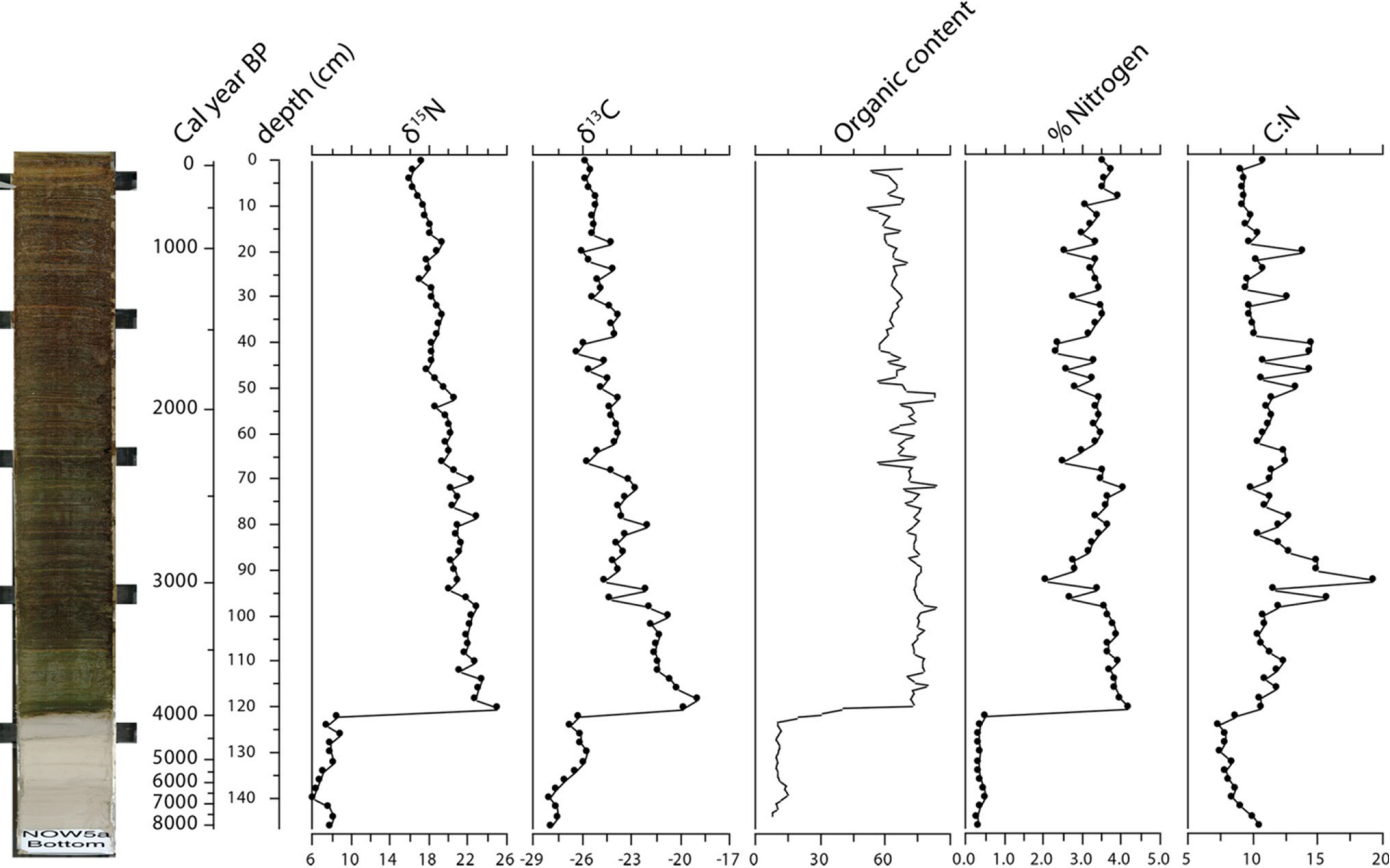
Sediment core collected from the lake within a large bird colony at Salve Ø. It shows the stepwise change in the characteristics of the lake ecosystem. The change was very rapid, showing a total change in sediment chemistry within less than a cm, corresponding to less than 30 years. The stable isotopes of C and N are in permille, organic content is loss of ignition in %, N is percent of organic matter and C:N ratio is calculated by weight (modified from Davidson et al. 2018)

The formation of the polynya itself may be a classic example of a tipping point (Box 1, Fig. 1b scenario). The North Water polynya relies upon the formation of the ice bridge or ice arc at the north end of Baffin Bay in Smith Sound in the Nares Strait. This stable ice bridge prevents pack ice from being blown south by the prevailing northern wind into Baffin Bay. In addition, new ice formed south of the ice bridge is blown further south, creating open water, and as dense salt water forms during ice creation, the dense salt water sinks and help creating entrainment of deeper water with nutrients to the photic zone. Lower spring temperatures would lead to the formation or persistence of the ice bridge allowing the North Water polynya to form. Therefore, a relatively small change in temperature might lead to a profound alteration of the environment and ecosystems (Box 1, Fig. 1b scenario). Davidson et al. (2018) suggest that the first arrival of the Little Auk at around 4400 cal years BP signals the genesis of the North Water polynya (Fig. 5). This coincides with falling temperatures at the end of the Holocene climate optimum; thus, it appears that a cooling climate counter-intuitively led to a longer period of open water and formation of large amounts of ice, but the ice was blown south, which resulted in the formation of the productive polynya. The effects of this relatively small change in temperature may have reverberated ever further in the ecosystem as the arrival of humans in the region is also first recorded at this time.

A sediment core from Salve Ø also revealed that the Little Auk arrival between 3945 and 3575 cal BP translated into rapid change in the terrestrial and aquatic ecosystems in the colonisation areas/catchments (Davidson et al. 2018). If Little Auk again disappears in the North Water Area, the bird-influenced systems will expectedly recover to their former state, though with a very significant delay (Box 1, Fig. 1d scenario). This is evident from a sediment core from Raffles Ø, off Liverpool Land, East Greenland, covering the past 10 000 years. Here, the seabird populations—mainly consisting of Little Auk—have varied substantially during the Holocene as judged from the concentration of cadmium, total nitrogen, and organic matter in the sediment. Large seabird breeding colonies were present between 7500 cal BP and 1900 cal BP, from 1000 to 500 cal BP and since circa 100 cal BP, their presence/absence being dependent on the feeding conditions close to Raffles Ø (sea-ice cover) and/or the degree of a sufficiently long breeding season due to climate conditions (Wagner and Melles 2001). In between these periods, the concentrations of total nitrogen and organic matter declined to pre-bird conditions, in this case, suggesting a relatively fast recovery.

## Discussion and conclusion: Uncertain futures and a socio-ecological system in transition

Discussing rapid transitions in the Arctic, notably in the North Water area, makes one realise the extreme complexity of the issue as has transpired from the cases described above. When and how do particular transformations qualify as transitions or even tipping points? Life in the multi-species community in the Thule Region has been sustained by the post-glacial polynya, which allowed both animals and humans to settle and remain there over four millennia—even if human settlement in the area was discontinuous (Hastrup et al. 2018). The hiatuses have a lot to do with climate fluctuations, as have the current changes, social and ecological, although there is no simple causality, as we have seen. In this section, we discuss current notions of fast transitions, resilience, and resistance with a view not only to past events but also to present and future prospects for the socio-ecological system. It underscores the need for human agents and political bodies to take action.

The Arctic is regarded as a sentinel of change, and significant changes in the climate system here are occurring faster than anticipated in the IPCC (2007) report (Carmack et al. 2012). This not only calls for an obvious natural scientific interest but also for a renewed attention to the people (still) living there, who are more or less dependent on a particular natural system for their livelihood. In addition, it calls for socio-ecological system analyses, certainly also in the North Water area. In the preceding historical cases, this has been exemplified in various ways. The challenge now is to be able not only to analyse the past but also to understand the future challenges.

Recognizing the North Water area as an integrated socio-ecological system prompts us to examine the present changes in the sea-ice system in relation to economic and political issues. While trying to curb the effects of the sea-ice changes, the political economy of predicting the future inadvertently contributes to the volatility and unpredictability of the system. “As we move further into the Anthropocene, the volatility of socio-ecological systems is increasing” (Young 2012, p. 75). Given the dramatic physical and biological changes, both public and private actors have taken new intense interest in shipping, industrial development, and tourism, intensifying the processes of environmental change. This affects the entire make-up of the Thule community. In the Thule region, part of the flexibility that has allowed people to live there over millennia, against all odds, has been their readiness to disperse and to shift between communal and more individualised family strategies based on the hunt. Resources were equally accessible to all, but not always in the same places as before.

The open-access nature of resources has been identified as a key feature of the so-called hunter-gatherer communities. Within this framework, it has been discussed when and how individual decisions about foraging strategies affect the entire forager-resource system—in turn feeding back on the decisions of individual foragers and potentially leading to stochastic environmental variation (Freeman and Anderies 2012, p. 439). While such abstract discussions of potential tipping points in a socio-ecological system may have their merits, they do tend to reduce the complexity of both the system and of human action. There is a lot more to human action than survival at the lowest possible cost-efficiency level; there are preferences and social relations, past experiences, and anticipated futures. Such is very much the case among the High Arctic hunters and their families living around the North Water. What is more, they are fully aware of the Anthropocene realities, and the challenges that the manifest environmental changes pose to their way of living. While it may be difficult to survive some periods, people are generally not averse to new futures (showing high resilience, Box 2). They may live in a relatively secluded region, but they are certainly abreast of both national and global developments (facing strong openness).

Within the social sciences, there is a certain weariness of the notion of tipping points, given that social and, indeed, socio-ecological processes are seen to have contributed to continual change of the surface of the earth in a process of more or less smooth or abrupt change. As Nuttall has it: “the world has been transformed by human action in a constant process of engagement between people and the environments they inhabit, forcing us to question the idea of the ‘natural world” (Nuttall 2012, p. 100). Even when the transformative human acts originate beyond the ‘natural system’ under scrutiny, because of the global impacts upon the North Water area, they are still part of the larger socio-ecological system with which we are concerned here—a ‘system’ that is made out of elements of many scales, temporal and spatial, and which is affected by material, technological, animal, and political processes originating elsewhere.

The hunting communities that have so far lived off the North Water’s resources of marine mammals, as well as available game on the coastal lands, such as reindeer (*Rangifer tarandus*) and muskox (*Ovibus moschatus*), not to speak of seabirds, are also acutely aware of the national and international protection schemes that no longer give them free access to game, undermining Freeman’s and Anderies’ foraging-effort model and the dynamics of hunter-gatherer intensification. Intensification is not an option; hunting is restricted by quotas on all the big marine mammals (except the seals) in the interest of (international) species protection and sustainable use of the living resources. This takes us back to the issue of governance, which is an important part of any socio-ecological system and decisive as far as the High Arctic system is concerned. “Governance is a social function involving the establishment and administration of assemblages of rights, rules, and decision-making procedures intended to steer socio-ecological systems towards pathways that are collectively desirable and away from pathways that are undesirable” (Young 2012, p. 78). The often-debated hunting management’s regimes, such as the induced quotas on marine mammals, are examples of such governance. This again alerts us to the slippery boundaries of the system, incorporating actors from far beyond the region—be they national agencies or global bodies, and ranging from the native Thule Law, in force from 1929 onwards, to UNCLOS, the United Nations Convention of the Law of the Sea.

Another ‘boundless’ source of impact upon the localised system immediately around the polynya is the changing ice cover in the entire region. The sea-ice, the ice arch and the coastal fast ice, is a defining element in the constitution of the North Water (Hastrup et al. 2018), and it forms the entire sea-ice community within and around it. This has been so ever since humans moved into the region some 4500 years ago. The dwindling fast ice has increasingly obvious repercussions on most species and on human–animal relations.

The polynya is an ice-free area at a time and a place where ice would be expected and where ice is being formed. The constant formation of ice and it being blown away are important for seeding the marine productivity that sustains the fish, marine mammals, birds, and humans of the region. In recent decades, the formation of the Ice arch has been more unreliable and the ice arch seems to break up earlier (Marchese et al. 2017). Warming in the region reduces ice formation in the Nares Strait, which cause instability of the ice bridge and eventually the loss of it and thus the polynya, and that will potentially have large and negative consequences for the primary production of what is currently the North Water area (Marchese et al. 2017). Counter-intuitively, in the short term, it will lead to greater ice cover with drifting pack ice in spring as more pack ice will be drifting south from the Arctic Ocean as it happened in 2007 (Rasmussen et al. 2011). However, alternatively, an ice arch may form and block the ice flow 500 km further north, as it has been observed in 2009 (Vincent 2013) changing the system in yet another direction. With increasing temperature in Baffin Bay, new fish species will move northwards, leading to a significantly higher predation on the copepods that the Little Auk relies upon, which, combined with reduced primary production, will likely result in a major decline in their population and thus a decline in the fuelling of the land ecosystems with marine-derived nutrients. Greening may, therefore, decline, likely with a long legacy as the nutrients are washed out (as seen earlier in East Greenland), but accelerated by more precipitation falling as rain in a warmer future. This means less food for consumers such as muskoxen. In the long term, however, the land will, with further warming, be colonised by additional plants (Normand et al. 2013), perhaps, compensating for the loss of primary production on land and in freshwaters due to a decline in the population of Little Auk.

While physical and biological transformations are expected, though still somewhat uncertain in the details, it is much less certain that they are adequate in explaining shifts in the socio-ecological system. The issues of politics, power, and governance rapidly enter into the equation and muddle the picture. The sea will open up for new, year-round shipping routes, tourism, and other encroachments on the region, which at first may seem to open up new opportunities for economic gains, but which may soon bypass the hunters’ and their families’ services due to the difference in scale of action. About 750 people live in the entire Thule Region—half of whom are children, and all of whom are pondering the future of their community. The changing climate incurs certain costs, but budgets are limited. So far, the Greenlandic Self-Rule Government has given extensive subsidies to hunters in need of bigger and more reliable motorboats, now that they have to reach hunting grounds that, until 5 or 10 years ago, could be reached on dog sledge. How far can the government subsidise a kind of living that is not economically viable?

The accelerated warming in the Arctic leading to rising costs of relief and investment in new climate-change proof infrastructures and other adaptive actions will soon make governments face a choice between a continuing support to increase the adaptive capacity of local, coastal communities, leaving them to their own devices or closing down some of the communities and move the inhabitants. Here, at the intersection between politics and economy, Huntington et al. (2012) suggest that another trade-off emerges between willingness and unwillingness to pay. “Regardless of who bears the cost, responding to climate change is not optional (e.g., Stern 2007). As the environment changes, society and its members will have to respond” (Huntington et al. 2012, p. 72). It may prove impossible not to change the socio-ecological system substantially. There is no way in which the attempts at redefining the Arctic as a natural reserve will effectively curb the threats from the outside (cf. Young 2012)—because the ‘outside’ is already implicated, and always has been, in the development of the North Water area.
